# College and Hospital Notes

**Published:** 1905-05

**Authors:** 


### COLLEGE AND HOSPITAL NOTES.

THE University of Buffalo, medical department, Alumni Association will hold its thirtieth annual meeting Wednesday and Thursday, May 3 and 4, 1905.

The alumni who have medical and surgical services at the following institutions have signified their willingness to present cases of interest: Buffalo General Hospital, Hospital of the Sisters of Charity, Erie County Hospital, State Hospital for the Insane, Children's Hospital, German Hospital, Emergency Hospital, Providence Retreat, German Deaconesses' Hospital Woman's Hospital, Riverside Hospital, Eye and Ear Infirmary, Charity Eye, Ear and Throat Infirmary, Dispensary German Hospital, Dispensary Emergency Hospital, Dispensary University of Buffalo.

The classes of '55,'65, '75, '85 and '95 will meet in reunion.

All members of the association are requested to register as soon as possible after arrival. The registry desk will be in the main hall of the college building, where the detailed program for the two days may be found; members of the reception committee will gladly answer any questions relating to the various clinics and other features of the program.

The executive committee again urges all alumni to enroll themselves as active members of the association. The advancement and success of the University is a benefit to each alumnus and he should show his loyalty to his Alma Mater by furthering the good work by his attendance, efforts and contributions.

PROGRAM.

*Wednesday, May 3, 1905.*

10 a. m. to 12 m.—Clinics will be held in some of the above named institutions.

1 p. m.—The resident alumni will entertain the out-of-town alumni at luncheon at the University Club, corner of Delaware avenue and Allen street. Please send word to the chairman of the executive committee if you will be present so that proper preparation may be made.

3 p. m. to 6 p. m.—Clinics will be held in some of the above named institutions.

8 p. m.—The business meeting of the alumni association will be held in Alumni Hall, at which time and place the president will deliver his annual address and scientific papers will be read and discussed. Following the business session the faculty of the medical department will give a smoker in the University library.

*Thursday, May 4, 1905.*

9 a. m.—Clinics will be held in some of the above named institutions.

11 a. m.—The annual commencement exercises will be held in the Teck Theatre during which an address to the graduating classes will be given by Rev. Richard Earle Locke.

1 p. m.—Drs. Matthew D. Mann, Roswell Park, Charles G. Stockton, Charles Cary, Irving M. Snow, Herbert U. Williams and DeLancey Rochester will entertain the members of the alumni association at luncheon at the Saturn Club, corner of Delaware avenue and Edward street. An acceptance as early as possible should be sent to Dr. DeLancey Rochester, 469 Franklin street, Buffalo.

3 p. m. to 6 p. m.—Clinics will be held in some of the above named institutions.

7 p. m.—The annual dinner will be held in the Iroquois Hotel. The committee has taken great pains to secure an admirable list of speakers for this occasion as well as some other extra features. Tickets for the dinner will be $2.50 each.

*Alumni Officers.*—President, DeLancey Rochester, '84, Buffalo; first vice-president, Fridolin Thoma, '87, Buffalo; second vice-president, Henry T. Benham, '90, Honeoye Falls; third vice-president, Jane W. Carroll, '91, Buffalo; fourth vice-president, G. A. Himmelsbach, '91, Buffalo; fifth vice-president, C. J. Reynolds, '90, Buffalo; treasurer, H. K. DeGroat, '97, Buffalo; secretary, Thomas H. McKee, '98, Buffalo.

*Trustees.*—A. W. Henchell, '89, 1909, Rochester; A. W. Bayliss, '89, 1908, Buffalo; Robert P. Bush, '74, 1907, Horseheads; D. W. Harrington, '71, 1906, Buffalo; Frank H. Moyer, '72, 1905, Moscow.

*Executive Committee.*—Albert T. Lytle, '93, chairman; Grover W. Wende, '89; Franklin W. Barrows, '93; DeLancey Rochester, *ex-officio*; Eli H. Long, *ex-officio*, all of Buffalo.

*Reception Committee.*—Marshall Clinton, '96, chairman, Buffalo.

---

THE Detroit College of Medicine will hold its thirty-seventh annual commencement Thursday, May 4, 1905, at 7.30 p. m., in the Light Guard armory. The JOURNAL acknowledges the courtesy of an invitation to attend the exercises.

---

THE College of Medicine, Syracuse University, has a four years' course of study. Its facilities for instruction are unusually good. They consist of a main building erected in 1896, containing a library of several thousand volumes, study and lecture rooms, and laboratories large, light, convenient and well equipped with the apparatus required in the study of the fundamental medical sciences. Its facilities also include access to three large hospitals and three institutions for purposes of clinical instruction.

---

McGILL University, Montreal, announces the tenth annual post-graduate course of the faculty of medicine from June 5 to June 30, 1905. Special courses are to be given and each post-graduate student may select those which he prefers, paying only the fee, in addition to matriculation, of the courses selected.

---

PROVIDENCE RETREAT, a private hospital for mental and nervous diseases, is about to erect a new building which will be upon the most approved modern plans. The institution is owned and controlled by the sisters of charity, subject to the supervision of the state commission of lunacy. The new building will be located directly in front of the old one at Main street and Humboldt parkway, Buffalo, and will have a frontage on Main street of 402 feet. The center building will have a frontage of 48 feet, and will be 86 feet deep and five stories high. The wings are to be four stories high. The buildings are to be of brick and stone, with a tile roof. The interior is to be fireproof, the smallest possible amount of wood being used in its construction.

### BUILT BY A FOOL.

Saint Bartholomew's Hospital, in London, in spite of the large subscriptions of the King, the Queen and of their friends, is once more in pecuniary difficulties, and the question is being seriously discussed of removing it to a much less costly site in the suburbs among more healthful surroundings. Few are aware that it was founded by Rahere, the famous court jester of King Henry I. It seems that while still a young man his conscience began to trouble him and led him to make a pilgrimage to Rome, where he fell ill, and being in fear of death made a vow that if he recovered he would build a hospital on his return. He did recover, journeyed home, and his intentions are said to have been confirmed by a vision of Saint Bartholomew, who pointed out Smithfield to him as the best site for his purpose.

It was a most unpromising place at that time, being outside the city walls, little better than a marsh, and with an unsavory reputation as the locale of hideous executions. However, it had the recommendation that its land was of no value, and King Henry, who probably regarded the whole affair as a joke, readily gave his jester a grant of the land.

Rahere set to work first to build the church and then a priory. Having no money to pay for the building, he adopted a singular expedient. Wearing his cap and bells, he started men carrying stone and mortar as a jest. The humor of the thing spread, and vast numbers joined in the joke of erecting a building under such conditions. Though constructed in this way at a minimum of cost, part of the original building, dating from 1102, remains intact to this day.

The original hospital was a part of the priory. On the dissolution of the monastery under Henry VIII., it passed to the crown, but at the instance of Sir Richard Gresham, then Lord Mayor of London, it was reëstablished as a hospital, with a royal endowment. Damaged by the great fire in the reign of King Charles II., it was rebuilt at the beginning of the eighteenth century, and has been added to since. Harvey, who discovered the circulation of the blood, was for thirty-four years physician there. —*Marquise de Fontenoy.*

---

The Council of Education of the American Medical Association held a meeting at Chicago, April 20, 1905, to which representatives from all sections of the country were invited to present views relating to medical education. Dr. A. Vander Veer, of Albany, presented the conditions prevailing in the state of New York. A report of the conference will be made to the house of delegates at Portland.

